# Mechanisms by Which Different Functional States of Mitochondria Define Yeast Longevity

**DOI:** 10.3390/ijms16035528

**Published:** 2015-03-11

**Authors:** Adam Beach, Anna Leonov, Anthony Arlia-Ciommo, Veronika Svistkova, Vicky Lutchman, Vladimir I. Titorenko

**Affiliations:** Department of Biology, Concordia University, 7141 Sherbrooke Street, West, SP Building, Room 501-13, Montreal, QC H4B 1R6, Canada; E-Mails: adam.pb.beach@gmail.com (A.B.); anna_leonova@yahoo.com (A.L.); anthony.arlia@outlook.com (A.A.-C.); klubnika_veronika@hotmail.com (V.S.); lutchman.david@hotmail.com (V.L.)

**Keywords:** yeast, cellular aging, longevity regulation, metabolism, interorganellar communications, mitochondria, mitochondrial respiration, mitochondrial membrane potential, mitochondrial reactive oxygen species, mitochondrial lipidome

## Abstract

Mitochondrial functionality is vital to organismal physiology. A body of evidence supports the notion that an age-related progressive decline in mitochondrial function is a hallmark of cellular and organismal aging in evolutionarily distant eukaryotes. Studies of the baker’s yeast *Saccharomyces cerevisiae*, a unicellular eukaryote, have led to discoveries of genes, signaling pathways and chemical compounds that modulate longevity-defining cellular processes in eukaryotic organisms across phyla. These studies have provided deep insights into mechanistic links that exist between different traits of mitochondrial functionality and cellular aging. The molecular mechanisms underlying the essential role of mitochondria as signaling organelles in yeast aging have begun to emerge. In this review, we discuss recent progress in understanding mechanisms by which different functional states of mitochondria define yeast longevity, outline the most important unanswered questions and suggest directions for future research.

## 1. Introduction

**Figure 1:**
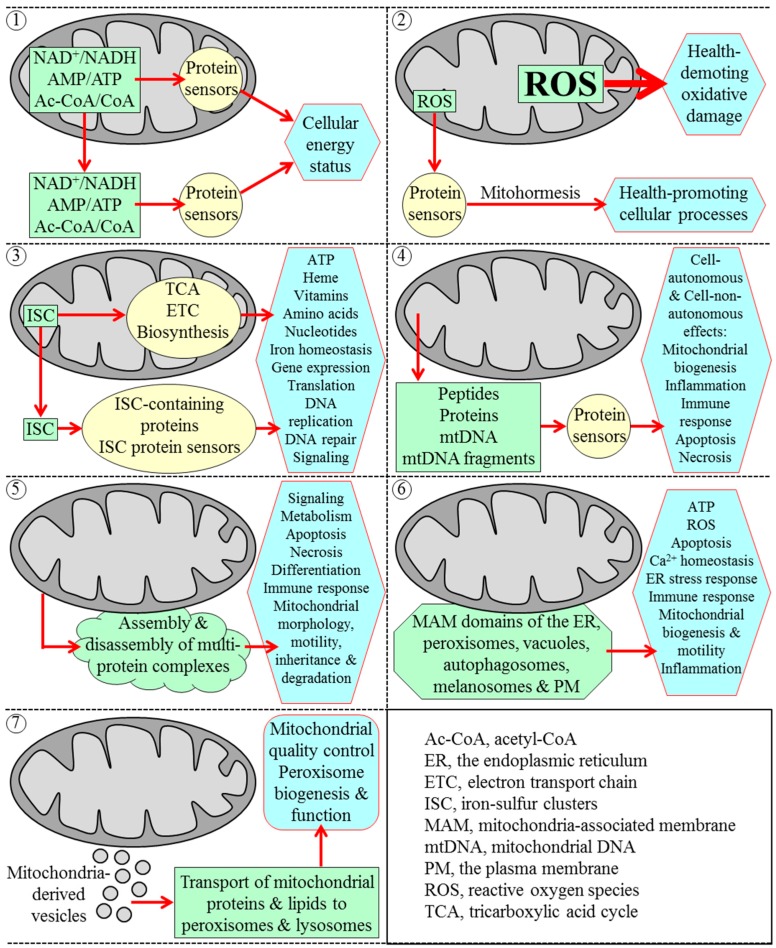
Seven strategies allow mitochondria to operate as intracellular signaling compartments and organizing platforms that orchestrate cellular responses to changes in physiological conditions. Strategy 1: mitochondria can change NAD^+^/NADH, AMP/ATP and acetyl-CoA/CoA ratios inside and outside of mitochondria, thus enabling these key metabolites to modulate activities of several protein sensors. These sensors then alter cellular energy status by amending activities of proteins involved in various metabolic pathways; Strategy 2: mitochondria can generate and release reactive oxygen species (ROS). If the concentration of ROS released from mitochondria surpasses a toxic threshold, ROS cause health-demoting oxidative damage to cellular macromolecules. In contrast, if it is maintained at a sub-lethal (hormetic) level, ROS modulate activities of several protein sensors, which then alter activities of many protein targets to accelerate health-promoting cellular processes; Strategy 3: mitochondria can synthesize and assemble iron-sulfur clusters (ISC). ISC are essential cofactors of numerous proteins located inside and outside of mitochondria; these proteins are involved in many cellular processes; Strategy 4: mitochondria can release peptides, proteins, mitochondrial DNA (mtDNA) and mtDNA fragments. These mitochondria-derived signaling macromolecules act in a cell-autonomous and cell-non-autonomous manner to elicit changes in vital processes that take place in the “host” cells and within the distal cells in other tissues; Strategy 5: mitochondrial surface can serve as an organizing platform for assembly and disassembly of multi-protein complexes implicated in a number of essential processes; Strategy 6: mitochondria can form zones of close apposition to the mitochondria-associated membrane (MAM) domains of other organellar and cellular membranes. These MAM domains are known for their essential roles in many cellular processes; Strategy 7: mitochondria can form small vesicles. These vesicles deliver certain proteins and lipids to lysosomes and peroxisomes, thereby enabling mitochondrial quality control and playing a role in peroxisome biogenesis and function. See the text for additional details.

The functional state of mitochondria is crucial for organismal physiology in all eukaryotes [1,2,3,4,5]. This is in part because mitochondria produce the bulk of cellular ATP and synthesize (or generate biosynthetic intermediates for) amino acids, lipids and nucleotides [3,4,5,6,7]. This is also because mitochondria can operate as intracellular signaling compartments and organizing platforms whose different functional states and dynamic communications with other organelles orchestrate a range of cellular responses to changes in various physiological conditions [1,3,5,7,8,9,10,11,12,13,14,15,16,17,18,19,20,21,22,23,24,25,26,27,28]. Several mechanistically diverse strategies have been implicated in enabling mitochondria to function as such signaling compartments and organizing platforms within eukaryotic cells; these strategies are depicted in Figure 1 and outlined below.

First strategy: mitochondria can respond to variations in cellular energy status by altering NAD^+^/NADH, AMP/ATP and acetyl-CoA/CoA ratios inside and outside of these organelles [3,23,29] (Figure 1). Such alterations in the relative levels of the key mitochondria-derived metabolites modulate activities of several protein sensors (including sirtuins Sirt1, Sirt3 and Sirt5 as well as the AMP-activated protein kinase and histone acetyltransferase GCN5), which then amend the extent of acetylation and phosphorylation (and thereby activities) of many downstream protein targets; this elicits a remodeling of a network of anabolic and catabolic pathways defining cellular energy status [3,19,23,29,30,31,32,33,34,35,36,37,38].

Second strategy: mitochondria can generate reactive oxygen species (ROS)—mainly as by-products of mitochondrial respiration in all eukaryotes, but also via oxidation of cytochrome *c* by the inter-membrane space protein p66^shc^ in mice [39,40,41] (Figure 1). If the concentration of ROS released from mitochondria exceeds a toxic threshold, ROS impair many vital cellular processes by eliciting health-demoting oxidative damage to proteins, lipids and nucleic acids in various cellular locations [39,40,41,42,43]. However, if the concentration of ROS released from mitochondria is sustained at a sub-lethal (hormetic) level, ROS operate as potent signaling molecules that modulate activities of some protein sensors [18,19,39,42,43,44,45,46,47]. Among these ROS-modulated sensors are several protein kinases, transcription factors and antioxidant enzymes; they respond to ROS by altering activities of a network of downstream protein targets to stimulate a number of vital health-promoting cellular processes [18,19,39,42,43,44,45,46,47,48,49,50,51,52,53].

Third strategy: mitochondria can synthesize and assemble iron-sulfur clusters (ISC); ISC are cofactors of many proteins that reside in mitochondria as well as in the cytosol, nucleus and endoplasmic reticulum (ER) [54,55,56,57,58,59] (Figure 1). Mitochondrial ISC proteins are essential components of the tricarboxylic acid (TCA) cycle and electron transport chain (ETC); they are also involved in the synthesis of some amino acids, heme, molybdenum cofactor, lipoic acid and biotin within mitochondria [54,55,56,57,58,59]. Cytosolic ISC proteins have been implicated in nucleotide metabolism, amino acid synthesis, iron homeostasis regulation, xenobiotic metabolism, translation initiation, tRNA modification and receptor tyrosine kinase signaling [54,55,56,57,58,59]. Nuclear ISC proteins function in DNA replication and repair, telomere maintenance, gene expression regulation, rRNA processing, and ribosome assembly [54,55,56,57,58,59,60,61,62,63].

Fourth strategy: mitochondria can respond to various stress conditions inside and outside of these organelles by releasing signaling peptides, proteins, mitochondrial DNA (mtDNA) and mtDNA fragments that act intracellularly and extracellularly to elicit cellular and organismal responses to such conditions (Figure 1). These mitochondria-derived stress signaling macromolecules include the following: (1) peptides formed due to the proteolytic degradation of unfolded and misfolded proteins excessively accumulated within the mitochondrial matrix; after being released from mitochondria, these peptides trigger several unfolded protein response (UPR^mt^) pathways of mitochondria-to-nucleus communications that stimulate transcription of nuclear genes involved in mitochondrial quality control and metabolism [18,19,53,64,65,66,67,68,69,70,71,72,73]; (2) humanin, an *N*-formylated peptide; after being synthesized and released by dysfunctional mitochondria in the “host” cells, humanin acts in a cell-autonomous manner to suppress apoptosis of these cells as well as in a cell-non-autonomous fashion to promote survival, reduce inflammation and maintain metabolic homeostasis of the distal cells in other tissues [72,74,75,76,77,78,79,80,81]; (3) signaling macromolecules that belong to the “mitochondrial damage-associated molecular pattern” (mitochondrial DAMP or MTD) family and include some *N*-formylated peptides, several proteins, mtDNA and mtDNA fragments; these MTDs function cell-autonomously and cell-non-autonomously to stimulate inflammation and elicit innate immune response by activating transcription of nuclear genes coding for pro-inflammatory cytokines [10,12,72,82,83,84,85,86,87,88,89]; (4) cytochrome *c* and other pro-apoptotic proteins; after being released from mitochondria in excessively stressed cells, these mitochondria-derived proteins either trigger caspase-dependent and caspase-independent pathways of programmed apoptotic cell death or control such non-apoptotic processes as cell metabolism, cell cycle progression, inflammation, innate immune response, autophagy and programmed necrotic cell death [3,9,10,12,13,16,17,23,24]; (5) the pyruvate dehydrogenase complex, which in cultured human cells exposed to serum, epidermal growth factor or mitochondrial stress can move from the mitochondria to the nucleus to synthesize acetyl-CoA used for histone acetylation, S phase entry and cell-cycle progression [90]; and (6) mitokines in the nematode *Caenorhabditis elegans*; after these diffusible factors of unknown chemical nature exit mildly stressed mitochondria in neuronal cells, they act in an endocrine-like fashion to activate the longevity-extending UPR^mt^ pathway of mitochondria-to-nucleus communication in intestinal cells [72,91,92].

Fifth strategy: the surface of mitochondria can provide an organizing platform for assembly and disassembly of several multi-protein complexes (Figure 1). A regulated remodeling of these protein complexes on the outer face of mitochondria has been implicated in many vital processes, including (1) cell metabolism, signaling, immune response, hypoxic response, differentiation and death; and (2) mitochondrial fusion, fission, motility, inheritance, autophagic degradation and DNA maintenance [1,3,9,14,16,21,22,23,24,26,93,94,95,96,97,98,99].

Sixth strategy: the mitochondrial outer membrane can form zones of close apposition to the mitochondria-associated membrane (MAM) domains of other organellar and cellular membranes, including the ER, peroxisomes, vacuoles, autophagosomes, melanosomes and plasma membrane [3,27,28,100,101,102,103,104,105,106,107,108] (Figure 1). The MAM domains are known to play essential roles in ATP and ROS production, mitochondrial biogenesis and morphology, mitochondrial fission and motility, ER stress regulation, cellular Ca^2+^ homeostasis, membrane phospholipids metabolism and transfer, autophagosome biogenesis, innate immune and inflammatory responses, and programmed apoptotic cell death [3,16,18,21,24,25,26,27,28,100,101,102,103,104,105,106,107,108,109,110,111,112,113,114,115].

Seventh strategy: mitochondria can form small vesicles that transport distinct subsets of mitochondrial proteins and lipids to peroxisomes and lysosomes (Figure 1). Such vesicular transport of mitochondria-derived protein and lipid cargos has been implicated in mitochondrial quality control; it may also play a role in peroxisome biogenesis and function [116,117,118,119,120,121].

Because mitochondrial functionality is vital to organismal physiology and health, mitochondrial dysfunction is a cause of numerous human mitochondrial disorders; these inborn disorders are due to mutations in nuclear genes and/or mtDNA, affect various tissues and organs, and exhibit a wide spectrum of clinical features [3,4,122,123,124,125]. Furthermore, mitochondrial dysfunction has been implicated in a variety of common human disorders, such as metabolic syndrome, obesity, type 2 diabetes, cardiomyopathies, neurodegenerative disorders and cancer [3,5,10,15,34,126,127,128,129,130,131]. Most of these common human disorders are aging-associated pathologies; moreover, a gradual deterioration of mitochondrial function is a distinctive and, likely, a causative feature of aging in eukaryotic organisms across phyla [2,10,18,19,132,133]. Therefore, an age-related progressive decline in mitochondrial function is considered as one of the hallmarks of cellular and organismal aging in evolutionarily distant eukaryotes [133]. Studies of the baker’s yeast *Saccharomyces cerevisiae*, a unicellular eukaryote, have provided deep insights into mechanisms linking mitochondrial functionality and cellular aging [2,18,19,20,134,135,136,137,138,139,140]. In this review, we discuss recent progress in understanding such mechanisms.

## 2. The Yeast *S. cerevisiae* Is a Beneficial Model Organism for Uncovering Mechanisms of Cellular Aging in Multicellular Eukaryotes

Aging of baker’s yeast is studied using robust assays. These assays are carried out under controllable laboratory conditions and measure two different aspects of the aging process in *S. cerevisiae*. Some of these assays monitor the replicative lifespan of yeast by measuring how many asymmetric mitotic divisions a mother cell could undergo before cell cycle arrest; replicative aging of yeast is believed to model aging of mitotic (*i.e*., capable of dividing) human cell types, such as fibroblasts, granulocytes, monocytes, lymphocytes and stem cells from amniotic fluid [137,141,142,143,144]. Other assays monitor the chronological lifespan of yeast by measuring how long a cell remains viable after cell cycle arrest; chronological aging of yeast is likely to mimic aging of post-mitotic (*i.e*., incapable of dividing) human cell types, such as mature neurons, adipocytes and mature muscle cells [137,143,145,146,147]. *S. cerevisiae* has relatively short replicative and chronological lifespans; moreover, it is amenable to comprehensive biochemical, molecular biological and cell biological analyses [137,142,146,148,149,150,151]. Because of these beneficial features of *S. cerevisiae* as a model for unveiling mechanisms of aging in multicellular eukaryotes, the use of the baker’s yeast has enabled to discover genes, signaling pathways and chemical compounds that govern longevity-defining cellular processes in eukaryotic organisms across phyla [18,19,20,137,143,144,146,147,150,152,153,154,155,156,157,158,159,160,161,162].

## 3. Mitochondria Are Signaling Organelles that Establish the Rate of Cellular Aging in Yeast by Orchestrating Many Processes Outside of Mitochondria

A body of recent evidence implies that alterations in certain traits of mitochondrial functionality early in life of replicatively or chronologically aging yeast impact many cellular processes outside of mitochondria, thereby orchestrating a stepwise development of a longevity-defining cellular pattern and its maintenance throughout lifespan. The molecular mechanisms underlying the essential role of mitochondria as signaling organelles in yeast aging have begun to emerge; they involve unidirectional and bidirectional communications between mitochondria and other cellular compartments. In this section, we discuss such mechanisms.

### 3.1. Mechanisms Underlying the Essential Role of Mitochondria in Yeast Replicative Aging

**Figure 2:**
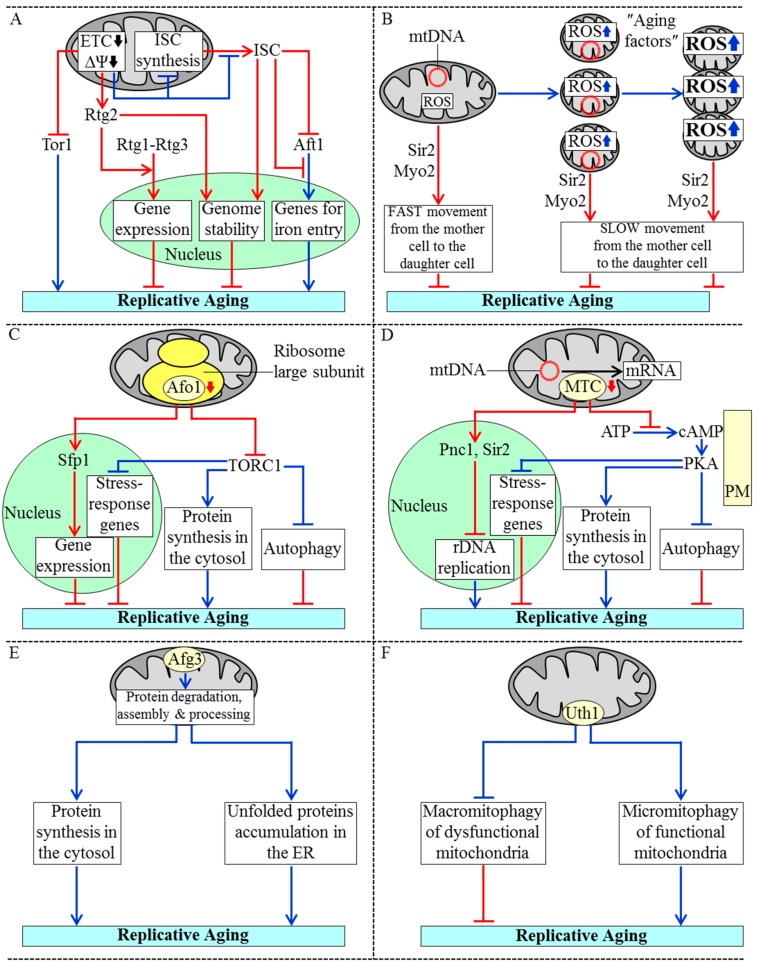
Mechanisms underlying the essential role of mitochondria in yeast replicative aging. (**A**) A decline in the mitochondrial electron transport chain (ETC) and the resulting reduction of mitochondrial membrane potential (ΔΨ) delay replicative aging in yeast by: (1) stimulating Rtg2-dependent nuclear import of the Rtg1-Rtg3 heterodimeric transcription factor, thereby activating the mitochondrial retrograde signaling pathway; (2) promoting the import of Rtg2 into the nucleus, where it increases genome stability; and (3) reducing a protein kinase activity of the target of rapamycin protein 1 (Tor1). The reduction of ΔΨ also attenuates mitochondrial synthesis and/or export of iron-sulfur clusters (ISC), which are needed for maintaining genome stability and inhibiting the Aft1-driven transcription of nuclear genes involved in iron entry into the cell; (**B**) A transition from the early-age stage to the intermediate-age stage of replicative aging coincides with a rise of mitochondrial reactive oxygen species (ROS) and fragmentation of mitochondria in the mother cell; these fragmented mitochondria produce more ROS, aggregate and lose mitochondrial DNA (mtDNA) during the subsequent late-age stage of replicative aging. The dysfunctional mitochondria formed during the intermediate-age and late-age stages of replicative aging are so-called “aging factors”. They limit the replicative lifespan of the mother cell; their Sir2- and Myo2-driven movement to the daughter cell occurs slower than that of fully functional mitochondria; (**C**) A reduction in the quantity of the Afo1 protein component of the large subunit of the mitochondrial ribosome delays replicative aging in yeast by: (1) stimulating Sfp1-driven transcription of nuclear genes encoding protein components of cytoplasmic ribosomes; and (2) attenuating the TOR signaling pathway, which accelerates the pro-aging process of protein synthesis in the cytosol and also slows down the anti-aging processes of autophagy and transcription of stress-response genes in the nucleus; (**D**) Mitochondrial translation control (MTC) proteins assist in processing, stabilization and translation of mRNAs encoded by mtDNA. A reduction in the quantity of MTC proteins slows down replicative aging in yeast by: (1) triggering the Pnc1- and Sir2-dependent inhibition of the synthesis of extrachromosomal rDNA circles; and (2) reducing the cellular concentration of cAMP and thus attenuating the cAMP-dependent protein kinase A (PKA) activity, which decelerates the anti-aging processes of autophagy and transcription of stress-response genes in the nucleus and also accelerates the pro-aging process of protein synthesis in the cytosol; (**E**) Afg3 is a mitochondrial protease involved in maintaining protein homeostasis (proteostasis) in mitochondria. The Afg3-driven processes in mitochondria include degradation of misfolded proteins, assembly of ETC protein complexes and processing of a protein component of the large subunit of the mitochondrial ribosome; these proteostatic processes within mitochondria stimulate such pro-aging processes outside of these organelles as protein synthesis in the cytosol and the accumulation of unfolded proteins in the endoplasmic reticulum (ER); (**F**) The Uth1 protein in the mitochondrial outer membrane is a pro-aging factor that accelerates replicative aging in yeast by concurrently weakening macromitophagic degradation of dysfunctional mitochondria and enhancing micromitophagic degradation of functional mitochondria. Activation arrows and inhibition bars denote pro-aging processes (displayed in blue color) or anti-aging processes (displayed in red color). See the text for additional details.

A mitotically competent mother yeast cell progresses through three consecutive stages of replicative aging before becoming unable to produce daughter cells; these stages are called “early age”, “intermediate age” and “late age” [20,144]. Characteristic features of the intermediate-age stage of replicative aging include a decline in mitochondrial respiration and the resulting reduction of mitochondrial membrane potential [20,144,163] (Figure 2A). A replicatively aging mother yeast cell responds to such reduction of mitochondrial membrane potential by activating the Rtg2 protein in the cytosol; a mechanism underlying such activation remains to be established [136,138,164]. Rtg2 is essential for reducing the rate of yeast replicative aging because this protein (1) triggers the mitochondrial retrograde signaling pathway by stimulating nuclear import of the Rtg1-Rtg3 heterodimeric transcription factor; this factor then orchestrates an anti-aging transcriptional program by activating expression of many nuclear genes involved in carbohydrate and nitrogen metabolism, peroxisomal fatty acid oxidation and anaplerotic reactions, peroxisome proliferation, stabilization of nuclear and mitochondrial genomes, and stress response [136,138,164]; and (2) is imported into the nucleus, where it increases genome stability and also slows down the synthesis of extrachromosomal rDNA circles known to be one of the “aging factors” limiting the replicative lifespan of the mother cell [136,138,164] (Figure 2A). The reduction of mitochondrial membrane potential during the intermediate-age stage of replicative aging slows down the aging process not only by activating the two Rtg2-driven mechanisms but also by inhibiting the target of rapamycin (TOR) complex 1 protein kinase activity [136,138,164] (Figure 2A); a mechanism of such inhibition of this key component of the pro-aging TOR signaling pathway is unknown. Moreover, the decline in mitochondrial membrane potential during the intermediate-age stage of replicative aging also decelerates mitochondrial synthesis of ISC and/or slows down their efflux from mitochondria [60] (Figure 2A). Because ISC are essential cofactors of nuclear proteins that in yeast are involved in DNA replication, DNA repair and telomere maintenance, their reduced quantity in replicatively aging yeast compromises genome integrity [18,20,60,62]. Moreover, because ISC mitigate activity and/or decelerate nuclear import of the transcription activator Aft1, their reduced quantity in replicatively aging yeast allows Aft1 to stimulate expression of nuclear genes needed for iron entry into and distribution within the cell; the ensuing rise in free intracellular iron accelerates replicative aging by promoting iron-driven oxidative damage of many cellular proteins [18,56,60].

During the intermediate-age stage of replicative aging, mitochondria in the mother cell not only exhibit reduced membrane potential but also generate elevated quantities of ROS and undergo fragmentation; during the subsequent late-age stage of replicative aging, the functionality of mitochondria is further declined as these organelles accumulate excessive levels of ROS, aggregate and fail to keep mtDNA [18,20,144,163,165,166,167,168] (Figure 2B). The dysfunctional mitochondria exhibiting these age-related traits of deteriorated functionality are known to be one of the aging factors limiting the replicative lifespan of the mother cell [20,137,141,144,161,163]. A mechanism exists for retaining these dysfunctional mitochondria within the mother cell, thus preventing their transmission into the daughter cell. In this mechanism, the dysfunctional mitochondria move from the mother cell to the daughter cell slower than their fully functional counterparts; such movement of mitochondria on actin cables is driven by the sirtuin Sir2 and type V myosin motor Myo2 [20,165,169,170,171] (Figure 2B).

The rate of yeast replicative aging is also modulated by the quantity of the Afo1 protein component of the large subunit of the mitochondrial ribosome; such modulation is not due to an effect of variations in Afo1 quantities on the efficiency of protein synthesis in mitochondria [2,18,172,173]. The absence of Afo1 in mitochondria of replicatively aging yeast elicits the following alterations in two signaling pathways of longevity regulation: (1) the anti-aging “back signaling” pathway is activated, thus enabling the Sfp1 protein to stimulate transcription of nuclear genes coding for protein components of cytoplasmic ribosomes [2,18,172]; and (2) the pro-aging TOR signaling pathway is inhibited, thus allowing to slow down the pro-aging process of protein synthesis in the cytosol and also to accelerate the anti-aging processes of autophagy, protein synthesis in mitochondria and transcription of many nuclear genes involved in cellular stress response [2,18,172] (Figure 2C).

Another group of mitochondrial processes that contribute to yeast replicative aging include the processing, stabilization and translational activation of mRNAs encoded by mtDNA; these processes are orchestrated by mitochondrial proteins constituting the so-called mitochondrial translation control (MTC) module [18,173,174,175,176,177] (Figure 2D). In the absence of any of these MTC proteins, the replicative aging of yeast is decelerated via the following two mechanisms: (1) the Pnc1- and Sir2-driven inhibition of the synthesis of extrachromosomal rDNA circles, one of the aging factors known to limit the replicative lifespan of the mother cell [18,176]; and (2) a decrease in the cellular concentration of cAMP and the ensuing attenuation of the cAMP-dependent protein kinase A (PKA) activity known to decelerate the anti-aging processes of autophagy and transcription of many stress-response genes in the nucleus as well as to accelerate the pro-aging process of protein synthesis in the cytosol [18,137,173,176] (Figure 2D).

The maintenance of protein homeostasis (proteostasis) in mitochondria also contributes to the essential role of these organelles as signaling compartments in yeast replicative aging [2,18,20,178,179,180]. One of the mitochondrial protein components implicated in maintaining mitochondrial proteostasis is the m-AAA protease Afg3. It is actively involved in several proteostatic processes within the mitochondrial inner membrane-including degradation of misfolded proteins, assembly of several ETC protein complexes and processing of a protein component of the large subunit of the mitochondrial ribosome [178,179] (Figure 2E). These Afg3-driven proteostatic processes in mitochondria increase the rate of yeast replicative aging, likely because of their demonstrated ability to promote such pro-aging processes outside of mitochondria as protein synthesis in the cytosol and the accumulation of unfolded proteins in the ER [180].

Finally, it seems that the quantity of the Uth1 protein in the mitochondrial outer membrane is inversely proportional to the efficiency with which only dysfunctional mitochondria exhibiting the age-related traits of deteriorated functionality are degraded within vacuoles of replicatively aging yeast. Indeed, it has been shown that lack of Uth1 (1) impairs micromitophagy, an autophagic mode of non-selective vacuolar degradation of both functional and dysfunctional mitochondria; and (2) does not affect macromitophagy, an autophagic mode of selective vacuolar degradation of only dysfunctional mitochondria [181,182,183]. Importantly, lack of Uth1 also extends longevity of replicatively aging yeast [181,182,183]. Thus, Uth1 may function as a pro-aging protein factor on the surface of mitochondria; any intervention that reduces its abundance is therefore expected to delay replicative aging in yeast by concomitantly enhancing macromitophagic degradation of dysfunctional mitochondria and weakening micromitophagic degradation of functional mitochondria [18,182] (Figure 2F).

### 3.2. Mechanisms by Which Mitochondrial Functionality Impacts Chronological Aging of Yeast

A culture of chronologically aging yeast in a liquid medium progresses through a series of critical lifespan periods that define its longevity; we have recently proposed to use the term “lifespan checkpoint” (or simply “checkpoint”) for describing each of these consecutive periods of yeast chronological lifespan [18,19,20,145,184]. The early-life checkpoints exist in logarithmic (L), diauxic (D) and post-diauxic (PD) growth phases; the late-life checkpoints take place in stationary (ST) phase of culturing [19,20,184]. Our concept of a “biomolecular network” posits that the rate and efficiency of progression through each of these lifespan checkpoints are monitored and/or modulated by a distinct set of checkpoint-specific “master regulator” proteins and protein complexes; a synergistic action of such master regulators defines longevity of chronologically aging yeast by orchestrating a stepwise development of a pro- or anti-aging cellular pattern [19,20,184]. A body of recent evidence supports the notion that different, age-related functional states of mitochondria define longevity of chronologically aging yeast by playing essential roles at several of the early-life and late-life checkpoints; we discuss this evidence below.

**Figure 3:**
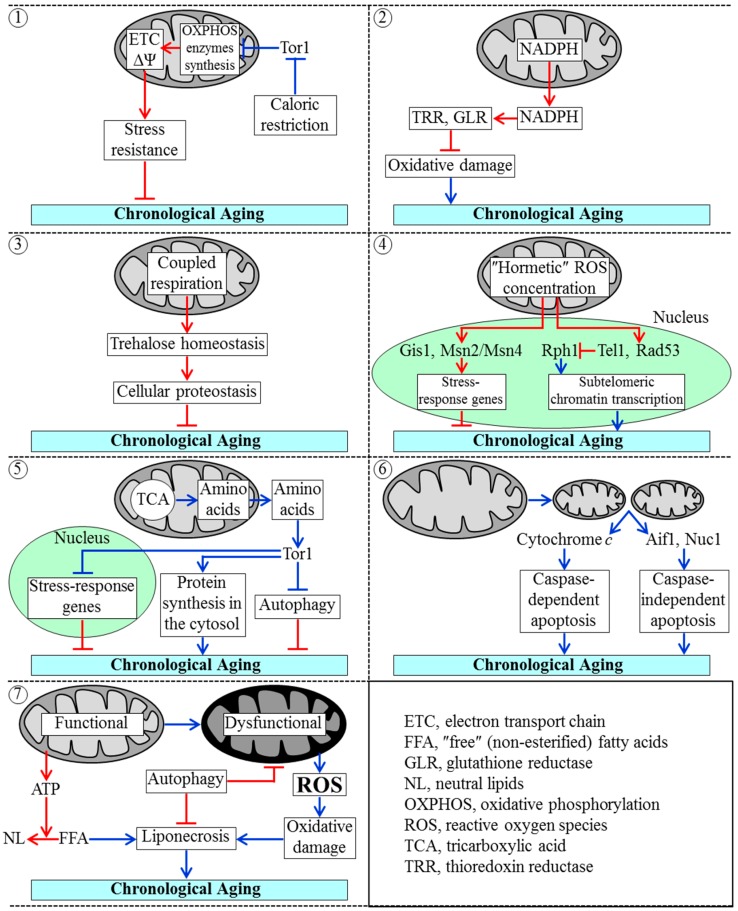
A chronologically aging culture of yeast cells progresses through at least seven consecutive “checkpoints”, each of which defines yeast longevity. Checkpoint 1: The protein kinase Tor1 mitigates the synthesis of mitochondrial DNA-encoded enzymes of the oxidative phosphorylation (OXPHOS) system in mitochondria, thereby allowing to set up a certain rate of electron flow through the mitochondrial electron transport chain (ETC) and a certain value of mitochondrial membrane potential (ΔΨ). These two traits of mitochondrial functionality establish a magnitude of cell resistance to stresses and, thus, define the chronological lifespan of yeast. Caloric restriction extends yeast chronological lifespan by suppressing Tor1; Checkpoint 2: Mitochondria-generated NADPH is an electron donor for thioredoxin and glutathione reductase (TRR and GLR, respectively) systems outside of mitochondria. Both these systems delay yeast chronological aging by reducing the degree of oxidative damage to many proteins in various cellular locations; Checkpoint 3: The efficiency of coupled mitochondrial respiration defines the cellular concentration of trehalose, which in chronologically “young” yeast cells acts as a longevity-extending metabolite by enhancing cellular proteostasis; Checkpoint 4: If mitochondria-generated reactive oxygen species (ROS) in chronologically “young” yeast cells are maintained at a “hormetic” level, ROS extend chronological lifespan by: (1) activating the transcription factors Gis1, Msn2 and Msn4, which promote expression of many longevity-extending nuclear genes; and (2) stimulating the longevity-extending Tel1-Rad53-Rph1 signaling pathway, which reduces the extent of damage to telomeric DNA in the nucleus; Checkpoint 5: Amino acids that are synthesized in and released from mitochondria activate the TOR signaling pathway; this pathway accelerates chronological aging by simulating protein synthesis in the cytosol, attenuating transcription of many stress-response genes in the nucleus, and suppressing autophagy of dysfunctional organelles and macromolecules; Checkpoint 6: A release of cytochrome *c*, Aif1 and Nuc1 from fragmented mitochondria in chronologically “old” yeast cells stimulates caspase-dependent and caspase-independent modes of apoptotic cell death, thus limiting yeast chronological lifespan; Checkpoint 7: Yeast chronological lifespan is shortened by “liponecrosis”, a mode of cell death that in chronologically “old” yeast cells is triggered due to (1) a reduced ability of mitochondria to generate ATP for the incorporation of non-esterified (“free”) fatty acids (FFA) into neutral lipids (NL); (2) a decline in the efficiency of an autophagic degradation of aged and dysfunctional mitochondria; and (3) a rise in the efficiencies with which mitochondria generate and release ROS. Activation arrows and inhibition bars denote pro-aging processes (displayed in blue color) or anti-aging processes (displayed in red color). See the text for additional details.

It seems that the earliest lifespan checkpoint defined by certain traits of mitochondrial functionality exists in L growth phase [19,20,145,155,184,185,186,187,188]; we call it “checkpoint 1”. At checkpoint 1, the rapamycin-sensitive protein kinase Tor1 (a key component of the pro-aging TOR pathway) attenuates mitochondrial synthesis of oxidative phosphorylation (OXPHOS) enzymes that are encoded by mtDNA [185,186,187,188] (Figure 3). The extent of such attenuation (1) is modulated by variations in cellular energy status; and (2) establishes a certain rate of coupled mitochondrial respiration and a certain value of mitochondrial membrane potential; both these key traits of mitochondrial functionality define longevity of chronologically aging yeast cells—in part by influencing stress resistance of such cells [19,20,145,155,185,186,187,188]. The rate of coupled mitochondrial respiration and the value of mitochondrial membrane potential at checkpoint 1 are also modulated by caloric restriction, a longevity-extending dietary regimen whose impact on these characteristic traits of mitochondrial functionality at checkpoint 1 is mediated in part by its inhibitory effect on Tor1 [19,20,137,155,185,186,187,188].

Mitochondria, along with the pentose phosphate pathway, generate NADPH [189,190,191]. In mitochondria of chronologically “young” yeast cells, this primary source of cellular reducing equivalents is produced in two reactions of the TCA cycle as well as in reactions catalyzed by an acetaldehyde dehydrogenase and an NADH kinase [189,190,191]. NADPH drives reductive reactions of the biosynthetic pathways for amino acids, fatty acids and sterols [189,190,191]; moreover, NADPH is also an electron donor for thioredoxin and glutathione reductase systems that maintain cellular redox homeostasis [189,191]. At lifespan checkpoint 2, which exists in D and PD growth phases, these two enzymatic systems play essential roles in delaying yeast chronological aging because they both reduce the extent of oxidative damage to many thiol-containing proteins in the mitochondria, nucleus and cytosol [19,20,191] (Figure 3).

The rate of coupled mitochondrial respiration in yeast cells progressing through D and PD growth phases defines their chronological lifespan in part by modulating cellular concentration of trehalose [19,20,145,188,192]. A lifespan checkpoint at which coupled mitochondrial respiration is linked to the maintenance of cellular trehalose homeostasis is called checkpoint 3 [19,20] (Figure 3). Trehalose is a non-reducing disaccharide that controls cellular proteostasis (*i.e*., a dynamic balance between protein folding, misfolding, unfolding, refolding, oxidative damage, solubility and aggregation) throughout lifespan of chronologically aging yeast [19,192].

A distinct lifespan checkpoint, which we call checkpoint 4, is defined by the concentration of ROS produced by and released from mitochondria in chronologically “young” yeast cells; this checkpoint exists in D and PD growth phases [19,20,139,140,145,193,194,195]. If the concentration of mitochondria-derived ROS in such cells is maintained at a hormetic level, these ROS act as signaling molecules that extend yeast longevity by: (1) stimulating the transcription factors Gis1, Msn2 and Msn4, which respond by activating expression of many nuclear genes known for their essential longevity-extending roles in nutrient sensing, carbohydrate metabolism, stress resistance and survival [140,194,195,196,197]; and (2) triggering the anti-aging Tel1-Rad53-Rph1 signaling pathway, which in response reduces the extent of damage to telomeric DNA by attenuating transcription of subtelomeric chromatin regions in the nucleus [140,194,195] (Figure 3).

The abilities of mitochondria to convert some of the intermediates of the TCA cycle into amino acids and to release these amino acids [189,190,198] define lifespan checkpoint 5, which exists in D and PD growth phases [18,19]. Following their release from mitochondria, neutral amino acids stimulate protein kinase Tor1; the ensuing activation of the pro-aging TOR signaling pathway accelerates chronological aging by promoting protein synthesis in the cytosol, attenuating protein synthesis in mitochondria, slowing down transcription of many nuclear genes involved in stress response, and hindering autophagic degradation of dysfunctional organelles and macromolecules [18,19,185,186,198,199,200,201,202,203] (Figure 3). Transcription of stress-response genes in the nucleus and autophagy in the cytoplasm are also attenuated by the pro-aging cAMP/PKA (cAMP/protein kinase A) signaling pathway, which overlaps with the TOR pathway [18,19,137,154,201,203].

The fragmentation of a tubular mitochondrial network into individual mitochondria and the efflux of some proteins (including cytochrome *c* as well as the “moonlighting” endonucleases Aif1 and Nuc1) from the fragmented mitochondria take place in chronologically “old” yeast cells that enter ST growth phase; these characteristic changes in mitochondrial morphology limit yeast chronological lifespan by eliciting the caspase-dependent and caspase-independent pathways of apoptotic programmed cell death (PCD) [19,145,155,204,205,206,207,208,209]. A lifespan checkpoint at which such age-related changes in mitochondrial morphology are linked to age-related forms of mitochondria-controlled apoptotic PCD exists in ST growth phase; we call it checkpoint 6 [19,20,145] (Figure 3).

A lifespan checkpoint at which several distinct traits of mitochondrial functionality and homeostasis are linked to an age-related “liponecrotic” form of PCD is called checkpoint 7; it exists in ST growth phase [19,20,210,211]. These traits include (1) the efficiency with which mitochondria generate energy needed for the detoxification of non-esterified fatty acids through their incorporation into neutral lipids; an age-related decline in such efficiency accelerates age-related liponecrotic PCD by causing the excessive accumulation of monounsaturated fatty acids in cellular membranes; (2) the efficiencies with which mitochondria produce and release ROS; an age-related rise in such efficiencies above a threshold accelerates age-related liponecrotic PCD by causing oxidative damage to cellular macromolecules and organelles; and (3) the efficiency with which aged and dysfunctional mitochondria undergo an Atg32- and Aup1-driven selective autophagic degradation; an age-related decline in such efficiency accelerates age-related liponecrotic PCD by impairing the maintenance of a healthy population of fully functional mitochondria [18,19,20,158,159,182,210,211,212,213,214,215] (Figure 3).

Some traits of mitochondrial functionality have not been linked to a particular lifespan checkpoint yet and may be associated with more than one of such checkpoints in chronologically aging yeast. One of these traits is the maintenance of mitochondrial genome integrity and copy number; the essential role of this trait in defining the rate of chronological aging depends on the mitochondrial base-excision repair enzyme Ntg1 [195]. It seems conceivable that an aging-associated mitigation of the Ntg1-governed pathway for maintaining mitochondrial genome integrity and copy number may underlie such longevity-shortening process observed in chronologically aging yeast as an age-related rise in the rates of mtDNA fragments migration to the nucleus and in their insertion into nuclear DNA [216,217]. The ensuing reduction of nuclear genome stability is an important contributing factor to chronological aging in yeast [18,19,195,216,217]. Of note, the Ntg1-governed pathway: (1) overlaps with a branch of the pro-aging TOR signaling pathway which at checkpoint 1 operates in attenuating mitochondrial synthesis of mtDNA-encoded OXPHOS enzymes; (2) modulates the anti-aging Tel1-Rad53-Rph1 signaling pathway which at checkpoint 4 responses to hormetic concentrations of mitochondria-generated ROS by decreasing the extent of damage to telomeric DNA [140,194,195] (Figure 3). These observations further support the notion that the Ntg1-dependent maintenance of mitochondrial genome integrity and copy number is a trait of mitochondrial functionality that may be associated with more than one of the lifespan checkpoints in chronologically aging yeast.

Another trait of mitochondrial functionality that may define more than one of the lifespan checkpoints in chronologically aging yeast is the lipid compositions of both mitochondrial membranes. Indeed, lithocholic bile acid (LCA) extends yeast chronological lifespan by eliciting an age-related remodeling of mitochondrial membrane lipidome [139,193,218]. Such remodeling is likely to be responsible for the age-related changes in mitochondrial size, number and morphology observed in chronologically aging yeast cells exposed to LCA, as well as for LCA-driven alterations in the age-related dynamics of mitochondrial respiration, membrane potential, ATP synthesis and ROS homeostasis seen in these cells [139,193,218]. Our recent data suggest that these characteristic changes in mitochondrial membrane lipidome and functionality elicited by LCA in chronologically aging yeast cells enable mitochondria to operate as signaling organelles that modulate activities of several transcription factors linked to some of the above lifespan checkpoints; these factors respond by altering transcription of many longevity-defining nuclear genes, thus establishing an anti-aging transcriptional program [219].

## 4. Conclusions and Future Perspectives

Studies of the yeast *S. cerevisiae* have revealed that mitochondria function as signaling organelles orchestrating many longevity-defining processes in other cellular compartments, thus establishing the rate of cellular aging. The molecular and cellular mechanisms by which various traits of mitochondrial functionality define longevity of replicatively and chronologically aging yeast have emerged. These mechanisms have been characterized separately for the replicative and chronological paradigms of yeast aging. These two paradigms are traditionally investigated under controllable laboratory conditions independently from each other by measuring two different aspects of the aging process in yeast. However, emergent evidence strongly suggests that the replicative and chronological modes of yeast aging are interconnected and may converge into a single aging process; for a recent review of this topic, see reference [20]. Therefore, the major challenge now is to understand how the longevity-defining traits of mitochondrial functionality and signaling pathways modulated by these traits in replicatively aging yeast influence longevity-defining cellular processes in chronologically aging yeast; and *vice versa*, how the longevity-defining mitochondrial functions and signaling pathways they control in chronologically aging yeast impact longevity-defining cellular processes in replicatively aging yeast.
